# Evaluation of Concrete Structural Cracking Behavior Induced by Early Drying Shrinkage

**DOI:** 10.3390/ma18020395

**Published:** 2025-01-16

**Authors:** Mengxi Zhang, Chuntian Lu, Qiaolin Min, Xinyue Wang, Yinpeng He, Genhua Deng, Yixin Wang

**Affiliations:** 1 State Key Laboratory of Hydroscience and Engineering, Tsinghua University, Beijing 100084, China; zhangmx@tju.edu.cn; 2 State Key Laboratory of Hydraulic Engineering Intelligent Construction and Operation, Tianjin University, Tianjin 300350, China; xinyuewang@tju.edu.cn (X.W.); hyp@tju.edu.cn (Y.H.); dgh_205163@tju.edu.cn (G.D.); yx_w@tju.edu.cn (Y.W.); 3 China Nuclear Power Engineering Co., Ltd. HeBei Branch, Shijiazhuang 050000, China; luct@cnpe.cc

**Keywords:** early drying shrinkage, cracking behavior, highly detailed simulation, ambient humidity, RCC dam

## Abstract

In this paper, the early drying shrinkage coefficients of different hydraulic cement mortars are calibrated through laboratory experiments for moderate-heat Portland cement (MHPC) and low-heat Portland cement (LHPC). By developing an improved mesoscale modeling approach, a 3D highly detailed simulation of concrete was generated, which incorporates the phases of mortar, aggregates, and interfacial transition zone (ITZ). The simulation result is in good agreement with the concrete early drying shrinkage experiment, exhibiting an error of less than 4.99% after 28 days. Subsequently, the mesoscale model is employed to explain the influence of the ambient humidity, cement type, and aggregate volume ratio on the early drying shrinkage performance of concrete. The results show that the early drying shrinkage coefficient of the LHPC is approximately 82% of the MHPC. Additionally, the depth of ambient humidity influence is about 15 mm from the concrete surface after 28 days. The early drying shrinkage can be controlled by increasing ambient humidity via the LHPC or raising the aggregate volume ratio. The mass-loss rate of concrete decreases as the ambient humidity or aggregate volume ratio increases during the process of drying shrinkage. Furthermore, the results quantify the influence patterns of various factors on drying shrinkage, thereby facilitating their application in assessing the cracking time induced by early drying shrinkage in roller-compacted concrete (RCC) dams. This provides theoretical guidance for crack prevention in concrete structures and aids in developing strategies for the construction of crack-free dams.

## 1. Introduction

The drying shrinkage of concrete structures is a primary concern of designers and operators in hydraulic engineering. The change in external humidity after hardening leads to a decrease in the concrete’s internal moisture, resulting in drying shrinkage [1,2]. The drying shrinkage deformation caused by inadequate curing of concrete after molding is a frequent engineering issue in constructing concrete structures. This issue will result in significant tensile stresses in the strong constraint zone of the structure, leading to premature cracks within the concrete and affecting the structural performance [3,4]. The drying shrinkage of concrete increases rapidly in the early stage and tends to be stable in the later stage, indicating that the early drying shrinkage has a significant impact on the mechanical response of concrete structures. Therefore, assessing concrete shrinkage in the early stage is crucial for determining structural cracking behavior.

In the past few decades, researchers have carried out plenty of laboratory experiments to explore the drying shrinkage characteristics of concrete. Wu et al. [5] found that cement types, fly ash content, auxiliary cementing materials, aggregates, and steel fibers have various effects on the shrinkage of high-performance concrete. Compared with natural aggregate, the shrinkage of recycled aggregate in concrete has a tendency to exhibit a higher shrinkage rate [6,7,8], indicating that the source of aggregates can impact the concrete shrinkage. The three-dimensional digital image correlation (3D-DIC) technique was employed to measure the strain and displacement fields of the aggregate concrete with various grain sizes during drying shrinkage, and it was revealed that coarse aggregates would enhance the non-uniformity of the strain field [9]. The exploitation of additives such as expansion agents, shrinkage-reducing agents, and super absorbent polymers can reduce the shrinkage of cementitious materials to varying degrees and efficiently decrease concrete shrinkage. Nevertheless, the early strength of concrete decreases due to the retarding effect of the shrinkage-reducing agent on the initial hydration reactions [10,11]. Xie et al. [12] put forward an effective shrinkage-reducing technology without considerable reduction of the concrete strength. For ultra-high performance concrete (UHPC), the shrinkage of concrete is essentially related to the shape and content of the fibers. The optimum fiber content to inhibit shrinkage of UHPC is 2% [13]. The study by Xia et al. [14] indicates that the compound of superabsorbent polymers and nano-silica positively affects the performance of alkali-activated concrete, providing an effective way to reduce drying shrinkage and maintain the strength of alkali-activated concrete. Yang et al. [15] found that the inclusion of soda residue substantially mitigates shrinkage and cracking in UHPC while also enhancing compressive strength. Samouh et al. [16,17] carried out drying shrinkage experiments on concrete specimens with different sizes in the presence of various levels of ambient humidity and found that the concrete drying shrinkage is independent of the concrete dimensions. When the drying shrinkage occurs the same throughout the concrete, the mass-loss rate grows with the lessening of the ambient humidity.

Physical tests allow for the direct determination of concrete drying shrinkage characteristics. Nevertheless, the preparation of concrete specimens necessitates significant labor and material expenses. This implies that drying shrinkage tests commonly take a lot of time, and it is difficult to examine the influence of each phase component on the properties of materials on a micro to macro scale. The numerical simulation methodologies are low-cost and typically exhibit strong repeatability [18,19,20], which can serve as an effective supplementary tool for physical tests. Idiart et al. [21] examined the influence of aggregate particle size and volume ratio on concrete drying shrinkage via a two-dimensional (2D) mesoscale numerical model by simplifying the concrete as aggregates and mortars. Havlásek et al. [22] carried out a numerical analysis of concrete shrinkage with different aggregate shapes and found that the existence of aggregate enhances the nonlinearity between humidity and concrete shrinkage. Tang et al. [23] proposed a numerical analysis model for evaluating the shrinkage performance of the concrete repair system. By generating rough interfaces with different fractal dimensions, the effects of bonding strength, aggregate, and roughness on the cracking and interface debonding for the repair system were investigated. Bolander et al. [24] employed the random lattice-based model to simulate the water transport in cement-based composites and demonstrated the crucial role of the interface zone between overlays and concrete substrates in structural performance. Ožbolt et al. [25,26,27,28,29,30] developed a 3D chemo-hygro-thermo-mechanical (CHTM) model and tested its applicability in various engineering scenarios, effectively predicting the mechanical properties of concrete structures. Li et al. [31] successfully prepared UHPC using Excel solver to optimize the Modified Andreason and Andersen model with a confirmed distribution modulus m = 0.23, and they found steel fibers (18.3–67.4%) substantially reduced plastic, autogenous, and drying shrinkage in UHPC. Nevertheless, simplifying the simulation model in the research poses challenges in accurately representing the model’s characteristics. It is necessary to perform a 3D highly detailed mesoscale simulation of the concrete drying shrinkage based on the actual aggregates.

Generally, the existing mesoscale numerical models of concrete include two approaches: continuous medium and discontinuous medium. The continuous medium considers concrete as a composite of aggregate, mortar, and interfacial transition zone (ITZ) [32,33]. This modeling method for the continuous medium is followed in two ways: (i) Actual mesoscale model [34,35,36,37], established based on X-ray computed tomography (XCT). Although it is accurate, the high expenses and excessive pretreatments make it difficult to be widely employed. (ii) Random mesoscale model [30,38,39,40,41,42], established based on the simplified aggregate random throwing algorithms. This approach has high efficiency, yet the aggregates appear predominantly spherical, ellipsoidal, or polyhedral, which cannot accurately reflect the actual shapes and extremely relies on aggregate throwing algorithms. The discontinuous medium approach is mostly utilized to simulate the flow, compaction, aggregate breakage, and irregular movement of fresh concrete. In this approach, the concrete mortar and aggregate are set equivalent to many rigid spherical particles or “particle clusters” with different particle sizes [43,44]. However, this approach is not yet mature enough to analyze the shrinkage of concrete drying.

In this paper, an improved, highly detailed mesoscale simulation method proposed by coupling continuous medium and discontinuous medium methods to perform the accurate evaluation of the concrete early drying shrinkage is proposed. Moderate-heat Portland cement (MHPC) and low-heat Portland cement (LHPC) were selected as the numerical study’s target materials because they are the most commonly used and widely applied construction materials in dam engineering, which have low-heat hydration properties and favorable crack resistance [45,46]. The drying shrinkage experiments for both MHPC and LHPC mortars are performed, and the drying shrinkage coefficients are calibrated using numerical simulations. The actual aggregate database is obtained through three-dimensional (3D) scanning technology, and the highly detailed mesoscale simulation model with mortar, aggregate, and ITZ is established. The numerical simulation of the concrete shrinkage deformation coupled with the humidity field is performed to examine the effects of ambient humidity, cement type, and aggregate volume ratio on the early drying shrinkage performance of concrete. The results are subsequently utilized in the evaluation of cracks in a roller compacted concrete (RCC) dam in the alpine region, which can provide strategies for the early maintenance of massive concrete structures and assist in the construction of crack-free dams.

This research introduces three significant innovations: First, it presents a refined simulation method for concrete that employs measured and calibrated data for key parameters, achieving high precision and efficiency in calculations. Second, it quantifies the influence of various factors on drying shrinkage, thereby providing a comprehensive understanding of their effects. Lastly, the study identifies the critical timeframe for drying shrinkage cracking in concrete, offering valuable guidance for construction practices.

## 2. Methodology

### 2.1. Overall Framework

**Figure 1:**
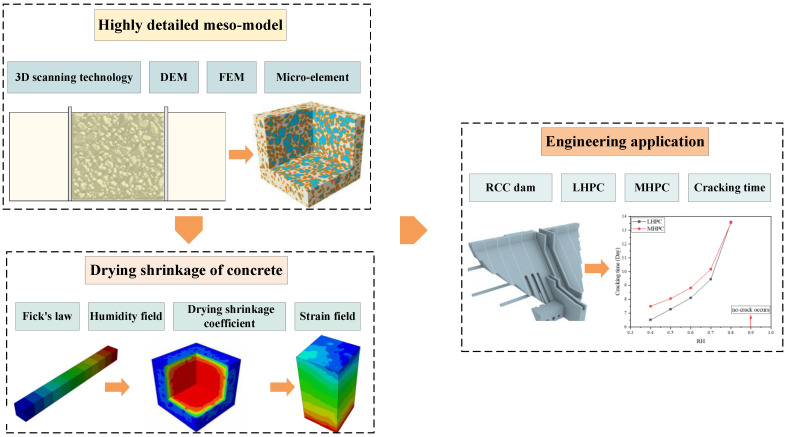
Overall research framework.

The overall research framework has been demonstrated in Figure 1. First, the highly detailed mesoscale model of concrete containing actual aggregates is established by coupling the discrete element method (DEM) and finite element method (FEM). Second, Fick’s law is implemented to predict the humidity field of concrete subjected to different levels of ambient humidity. Based on the time-varying model of the drying shrinkage coefficient, the strain field of the concrete is analyzed, and the drying shrinkage deformation of concrete due to various factors is obtained. Finally, the simulation results are applied to the RCC dam project, and a method for reducing the concrete drying shrinkage of massive concrete structures is proposed.

### 2.2. Humidity Field of the Concrete

The hygro-mechanical (HM) model formulated employs the diffusion equation (Fick’s law) with the effect of self-desiccation for the moisture transport [47,48]. The variation law of the humidity field in concrete can be described in differential form as Equation (1), and the theoretical model of the humidity field is established.

(1) $$\frac{\partial H}{\partial t}=D(\frac{\partial^{2}H}{\partial x^{2}}+\frac{\partial^{2}H}{\partial y^{2}}+\frac{\partial^{2}H}{\partial z^{2}})-g(t)$$

where H presents the relative humidity in the concrete at time *t*; D is the moisture diffusivity, mm^2^/day; g(t) denotes the humidity loss rate function of concrete in the drying process due to the self-desiccation process, which can be calculated with Equation (2) [49,50].

(2) $$g(t)=\frac{hk_{z}}{{\left(1+k_{z}t\right)}^{1.2}}$$

where $k_{z}$ is a constant, taken as 0.42637 day^−1^; t is the time, day; h is a function related to the water-cement ratio of concrete, which can be calculated according to Equation (3).

(3) $$h=0.00478e^{-\frac{w/c}{0.3068}}$$

where w/c is the water-cement ratio of the concrete, which is 0.5 in this paper.

The boundary condition of the humidity field in the concrete drying shrinkage is shown in Equation (4).

(4) $$-D\frac{\partial H}{\partial n}=\beta (H_{m}-H_{s})$$

where $H_{m}$ denotes the humidity of the material surface; $H_{s}$ represents the ambient humidity; β is the humidity exchange coefficient. The humidity diffusion coefficient, D, is related to the current humidity [51], and the moisture diffusion coefficient inside concrete can be estimated by:

(5) $$D=\frac{1.392{\left(1.0035-H\right)}^{0.5}+0.816H-0.888}{{\left(1.0035-H\right)}^{1.5}}$$

where H is the humidity inside the concrete at the current moment; D denotes the humidity diffusion coefficient, mm^2^/day. It is emphasized that the concrete is a multiphase composite structure consisting of mortar, ITZ, and aggregates. Due to the porosity of the ITZ, the humidity diffusion coefficient is higher than that of the mortar and is defined by $D_{ITZ}=10D$ [52]. Further, the aggregate’s diffusivity is about $\frac{1}{50}$ times the mortar’s diffusivity, i.e., $D_{st}=\frac{1}{50}D$ [53].

### 2.3. Strain Field of the Concrete

The strain field of concrete can be evaluated from the distribution of the humidity field [53], and finally, the drying shrinkage deformation of concrete can be calculated as follows:

(6) $$\Delta \varepsilon_{sh}=\alpha_{sh}\Delta H$$

where $\Delta \varepsilon_{sh}$ represents the strain change during the drying process; $\alpha_{sh}$ denotes the drying shrinkage coefficient; ΔH is the humidity change. Due to the porosity of the ITZ, the drying shrinkage coefficient is higher than that of the mortar and is defined by $\alpha_{sh,ITZ}=2.28\alpha_{sh}$ and the drying shrinkage coefficient for aggregate is 0 [52].

## 3. Early Drying Shrinkage Performance of Mortar

### 3.1. Mortar Drying Shrinkage Experiments

JC/T 603-2004 standard [54] is employed to test the drying shrinkage deformation of mortar. The dry sample is formed by a three-step die with dimensions of 25 mm × 25 mm × 250 mm. The water-cement ratio is 0.5, and the ratio of cement to sand is 1:3. The specimens are demolded within 24 h after molding and then are cured in water at 20 °C for 2 days. Each specimen is taken out, cleaned, measured for early growth, and subsequently placed in a curing box with constant temperature and humidity control (the temperature and relative humidity are kept at 20 °C ± 1 °C and 50% ± 3%, respectively). The length of the specimen is measured at each age (*l_t_*), and thereby, the drying shrinkage of the mortar at age *t* can be evaluated as follows:

(7) $$L_{t}=\frac{l_{0}-l_{t}}{l_{0}}\times 100\%$$

where *L_t_* is the drying shrinkage of the mortar at age *t*; *l*_0_ is the initial length of the specimen.

The MHPC and LHPC are employed to prepare mortars for drying shrinkage experiments. The main chemical compositions of the MHPC and LHPC are listed in Table 1, and the drying shrinkage test results of different mortars are provided in Table 2. The results demonstrate that at 3 days, the drying shrinkage of MHPC is comparable to that of LHPC; however, at 28 days, the drying shrinkage of MHPC is significantly higher than that of LHPC. The drying shrinkage of MHPC increases by 21% compared to LHPC. This increase may be related to its higher CaO content, which accelerates the hydration reaction and depletes the internal moisture of the mortar, leading to a greater drying shrinkage in the specimens.

### 3.2. Calibration of Drying Shrinkage Coefficient

**Figure 2:**
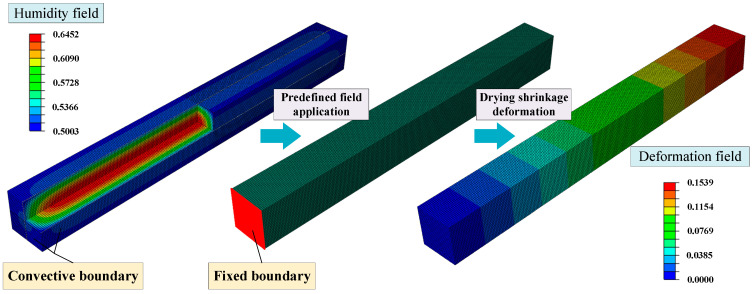
The simulation model of the mortar.

The simulation model for the mortar is established with hexahedral elements in ABAQUS 2022. The total numbers of elements and nodes in the model are 156,250 and 169,676. In the simulation of the humidity field, the initial humidity inside the concrete is 1, and the six surfaces of the model are assumed to be subjected to convection exchange conditions. The coefficient of surface humidity exchange is 1.5 mm/d [53], and the ambient humidity is 0.5, which is consistent with the experiment. The mortar drying shrinkage deformation is simulated by coupling the calculated humidity field, and the model is allowed to deform freely in the longitudinal direction, as demonstrated in Figure 2. Due to the high water-cement ratio and absence of silica fume in the experiment, the influence of autogenous shrinkage can be neglected after 28 days of curing according to the code (ASTM C1698-19) [55].

**Figure 3:**
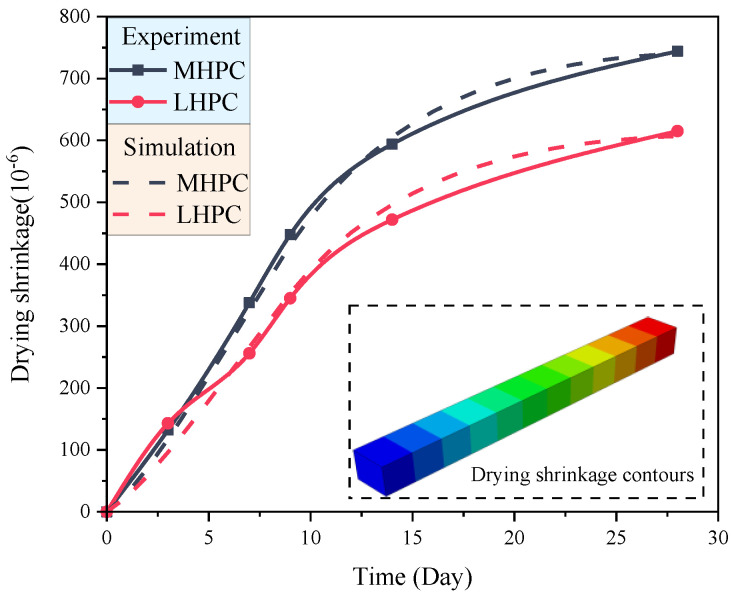
Early drying shrinkage of the mortar.

From the experimental data, calibration is performed on the early drying shrinkage coefficients of different mortars, and the drying shrinkage of the MHPC and LHPC mortars in 28 days is obtained. The results obtained in the simulation are consistent with those of the experiment, as shown in Figure 3. The calibration procedures for drying shrinkage coefficients begin by giving an initial value of drying shrinkage coefficients (0.0168/d), which is the statistical value of the concrete coefficient. Then, the drying shrinkage is simulated, and the root mean square error of experimental and simulation data can be obtained. In this case, the calibration process can be considered an optimization problem, considering the error to be the objective function. The time-varying drying shrinkage coefficients of different mortars are constantly adjusted and iterated to minimize the root mean square error of experimental and simulation data. Then, the early drying shrinkage of the MHPC and LHPC mortars is obtained. As illustrated in Figure 3, the predicted results by the numerical model are consistent with those of the experiment. Furthermore, the time-varying drying shrinkage coefficients of the MHPC and LHPC mortars in 28 days satisfy the corresponding relations as presented in Equations (8) and (9), and the drying shrinkage coefficient of the LHPC mortar is 82% of MHPC mortar.

(8) $$\alpha_{sh,M}=\left\{\begin{array}{c} 0.0004t,\;t\leq 9\\ -1.63\times 10^{-5}t+0.00507,\;9<t\leq 28 \end{array}\right.$$

(9) $$\alpha_{sh,L}=0.82\left\{\begin{array}{c} 0.0004t,\;t\leq 9\\ -1.63\times 10^{-5}t+0.00507,\;9<t\leq 28 \end{array}\right.$$

## 4. Establishment of Highly Detailed Concrete Mesoscale Model

Highly detailed simulation has gradually become the trend of concrete property analysis, which puts forward higher requirements for concrete mesoscale simulation [56,57,58,59,60]. It is mainly reflected in the following aspects: (i) Concrete is no longer treated as a single medium in the mesoscale model, and the distribution of microscopic materials such as aggregate, mortar, and ITZ should be considered in simulation; (ii) Concrete mesoscale model should describe the geometric features of the actual aggregate in detail; (iii) Concrete mesoscale model must ensure the quality of the mesh, simulation accuracy simultaneously. To meet these requirements, we perform thorough research.

### 4.1. Generation of Actual Aggregates

Currently, the majority of aggregates in the concrete mesoscale simulation are represented as simplified spheres, ellipsoids, or polyhedrons, which fail to accurately reflect the actual shape, and this limitation largely depends on the algorithm of the aggregate throwing process. Efficiently conducting mesoscale simulations for concrete is still a significant challenge. To address this issue, this paper improves it through the following procedures.

Step 1: Geometric model establishment of actual aggregates. The typical aggregates in engineering practice are selected to obtain the point cloud data using 3D scanning technology. According to the reverse modeling method, the geometric model of aggregate composed of multiple triangular surfaces is established, which can reflect the shape of actual aggregates. Subsequently, the database of actual aggregates employed in engineering is established.

Step 2: Actual aggregate filling with spherical particles. The geometric model of actual aggregate is filled with spherical particles to generate a “particle cluster”, and the appropriate number and size of particles can accurately reproduce the geometric characteristics of the actual aggregate. Based on the interactions between particles in the discontinuous medium, the position of the final “particle clusters” can be calculated.

Step 3: Generation of actual aggregates in the discontinuous medium. The actual aggregates in the space domain are generated in the following manner. First, based on the discontinuous medium method, the actual aggregate “particle clusters” with different volume ratios are generated in a large cubic space (the size can be flexibly adjusted according to the calculation requirements). In order to ensure the filling effect of the aggregate, the geometric parameters of “particle clusters” for actual aggregates are enlarged by 1.05 times, and an extrusion plate is set at the boundary in the length direction of the cube. Second, the extruded plate at the boundary uniformly shrinks inward until the aggregate shrinks to within 150 mm.

Step 4: Generation of highly detailed aggregates in the continuous medium. The aggregate size, position, and rotation information in the previous step are extracted, and the aggregate geometric model is mapped to the continuous medium. Therefore, the highly detailed geometrical model of aggregates for concrete mesoscale simulation is generated.

### 4.2. Volume Ratio of the Micro-Element

Concrete can be regarded as a three-phase composite material (aggregate, mortar, and ITZ). The traditional method of establishing the mesoscale model of irregular actual aggregates is faced with problems such as poor mesh quality and difficulty in convergence. Additionally, the geometric model of aggregate has many triangular surfaces with 3D scanning technology. When the mesoscale simulation model is established with free mesh, tetrahedral elements with small mesh widths will be formed in the sharp corners, which greatly affects the efficiency of the model. To solve the problem, the mesoscale simulation model is appropriately divided into regular hexahedral micro-elements with a length of 1 mm. It is possible to identify the aggregate, mortar, and ITZ by computing the volume ratio of each micro-element, thereby avoiding the meshing process of aggregates. The calculation method of the micro-element volume ratio can be simply given by:

(10) $$\rho =\frac{V_{A\cap B}}{V_{B}}$$

where ρ represents the volume ratio of the micro-element; $V_{A\cap B}$ is the volume of the intersection area between the micro-element and the aggregate, mm^3^; $V_{B}$ is the micro-element volume (herein, set equal to 1 mm^3^). Considering the cost of calculation and efficiency, the thickness of the ITZ is established at 1 mm, which satisfies the accuracy requirement [57,61,62,63]. It is worth noting that the developed model generation approach coupled the continuous and discontinuous media methods to construct a concrete meso-model. The mesh size can be effectively controlled, which is suitable not only for cubic concrete modeling but also for concrete models with different shapes. This method shows favorable applicability in corrosion cracking of reinforced concrete, and there is no need to mesh the geometric model of aggregate to ensure the mesh quality and calculation efficiency.

**Figure 4:**
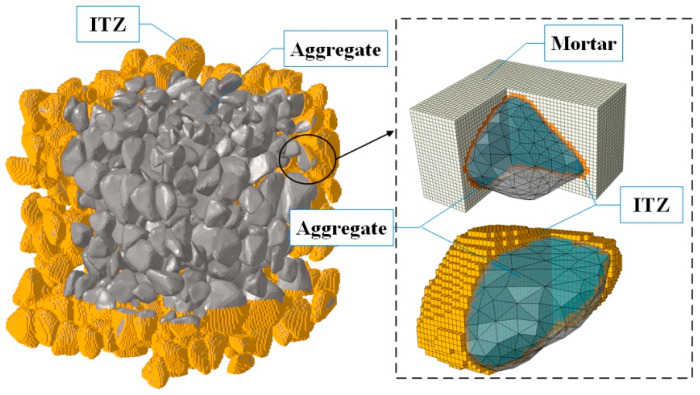
Mesoscale model of concrete based on the volume ratio of the micro-element method.

By implementing the calculation method for the volume ratio of regular micro-elements mentioned above, the width of elements can be controlled, and the phase of elements in the concrete mesoscale model can be quickly distinguished and combined. Figure 4 shows the aggregate distribution generated from the real aggregate morphology using Steps 1 to 4, outlined in Section 4.1. Firstly, the geometric model of aggregate is established by the 3D scanning technology. Then, the geometric model is filled with spherical particles and the position of the final particles. Thirdly, particles are placed and compressed in a discontinuous medium. Finally, The information aggregate is extracted and mapped to the continuous medium, as shown in Figure 4. Based on the volumetric method, 1 mm-sized hexahedral units of the interfacial transition zone (ITZ) are generated. Subsequently, aggregates and the mortar matrix are represented using 1 mm hexahedral units to establish a detailed mesoscale model of the concrete. This model distinctly differentiates and reflects the aggregates, mortar, and ITZ, ensuring the geometric characteristics of the actual aggregates are preserved and avoiding inaccuracies that may arise from simplified spherical or polyhedral aggregates. In the calculation of concrete shrinkage, the mass-loss rate can be quickly evaluated.

### 4.3. Mesoscale Model of Concrete

**Figure 5:**
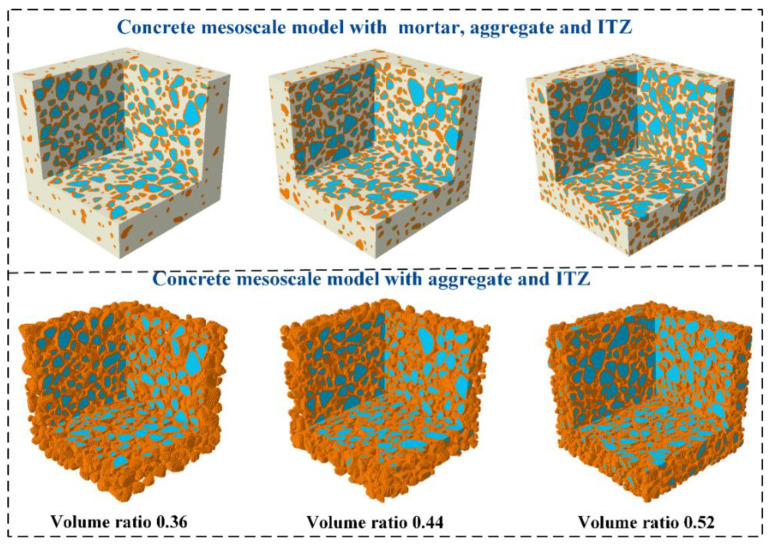
Mesoscale models of concrete with different aggregate volume ratios.

The aggregate content of hydraulic concrete is higher than that of ordinary concrete. Herein, the concrete aggregate with two grades is implemented in the mesoscale model. The volume ratios of aggregate are 0.36, 0.44, and 0.52, and the corresponding aggregate particles are 1702, 2271, and 2711, respectively. Through employing the above method, a mesoscale simulation model of concrete based on the actual aggregate in the continuous medium is established. The corresponding mesoscale models of concrete mortar, aggregate, and ITZ have been illustrated in Figure 5. The standard cube concrete is used for the calculation of the concrete internal humidity field, and the calculation method of the concrete mass-loss rate *m* during drying shrinkage is given by:

(11) $$m=\frac{\sum (1-RH)\gamma_{w}V_{B}}{\sum (\gamma_{w}+\gamma_{B})V_{B}}$$

where *m* denotes the mass-loss rate; RH represents the corresponding humidity of each element; $\gamma_{w}$ is the weight of water; $\gamma_{B}$ is the weight of concrete; $V_{B}$ is its corresponding volume. To increase the efficiency of calculating the drying shrinkage deformation field, a quarter of the standard cube and enforcing the axisymmetric boundary condition on the relevant sections are implemented.

### 4.4. Material Properties

The mesoscale simulation model has the ability to reproduce the 28-days compressive and tensile strength of concrete [64]. In the analysis for the concrete drying shrinkage, the properties for each phase in the mesoscale simulation model are listed in Table 3.

## 5. Concrete Early Drying Shrinkage Characteristics

### 5.1. Concrete Internal Humidity Field

**Figure 6:**
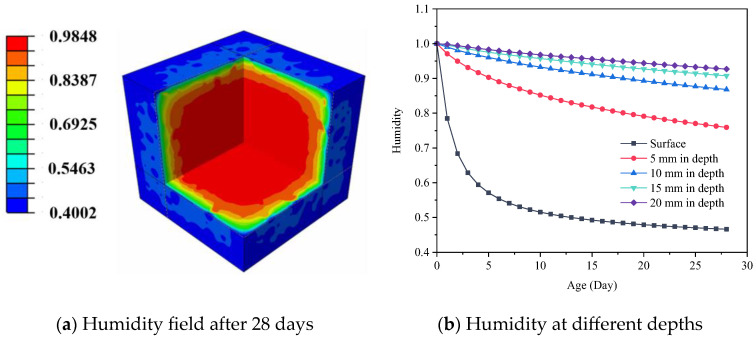
Concrete internal humidity field.

The concrete internal humidity field is shown in Figure 6. The result demonstrates that the humidity near the surface of the concrete decreases gradually, and the humidity at the center of the concrete is higher after 28 days. According to the statistics of the axial humidity changes of standard concrete specimens, it can be seen that the surface humidity of early concrete decreases faster than the internal humidity of concrete [65]. Furthermore, the humidity of concrete gradually increases with the growth of depth, indicating that the humidity of concrete gradually diffuses from outside to inside during the drying process.

**Figure 7:**
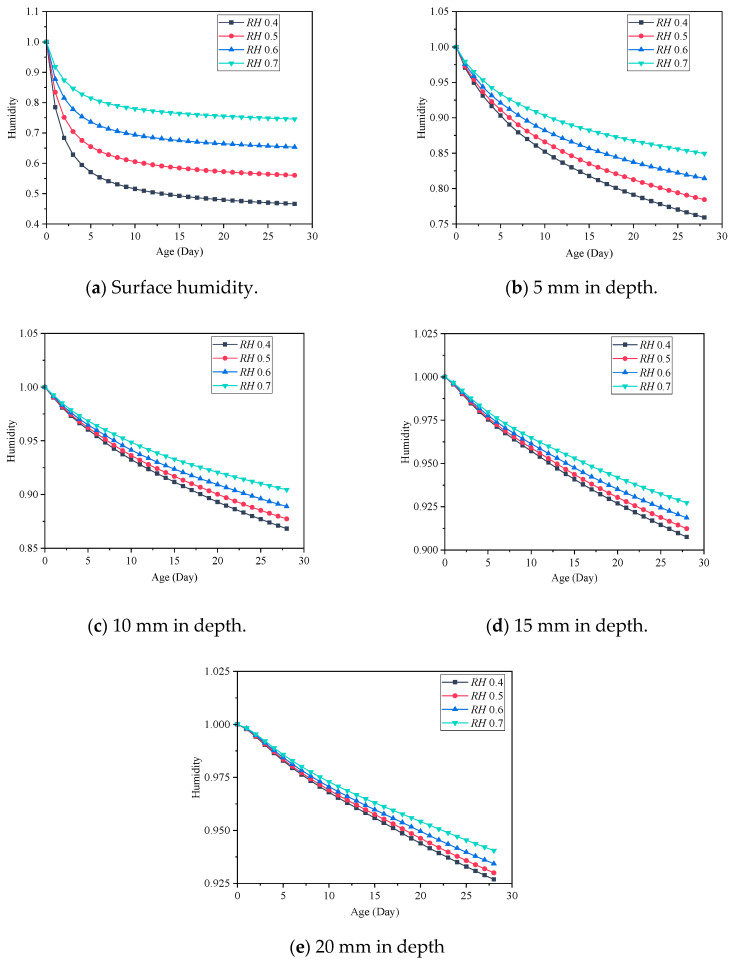
Concrete internal humidity under different ambient humidity.

The variation of concrete internal humidity in the presence of different levels of ambient humidity has been demonstrated in Figure 7. It can be found that at the same depth of concrete, the internal humidity of concrete with low ambient humidity decreases rapidly. On the surface of the specimens, humidity rapidly decreases from 1 to a plateau phase within 7 days, followed by a slow decline. However, the internal humidity of the specimens exhibits a linear decreasing trend, which is inconsistent with the decline observed on the surface. The results also indicate that the surface humidity of the specimens remains consistently higher than the ambient humidity, reflecting the transport of internal moisture to the surface. When the depth changes from 5 mm to 10 mm, the internal humidity increases from 0.75 to 0.875. When the depth reaches 15 mm, the internal humidity of the concrete remains above 0.9 for 28 days in low ambient humidity. However, below the depth of 15 mm, significant variations in internal humidity are observed, suggesting that the influence range of concrete humidity in the early stage is within 15 mm from the surface, which is consistent with the experimental results of Samouh et al. [16,17].

### 5.2. Verification of Concrete Drying Shrinkage Experiments

**Figure 8:**
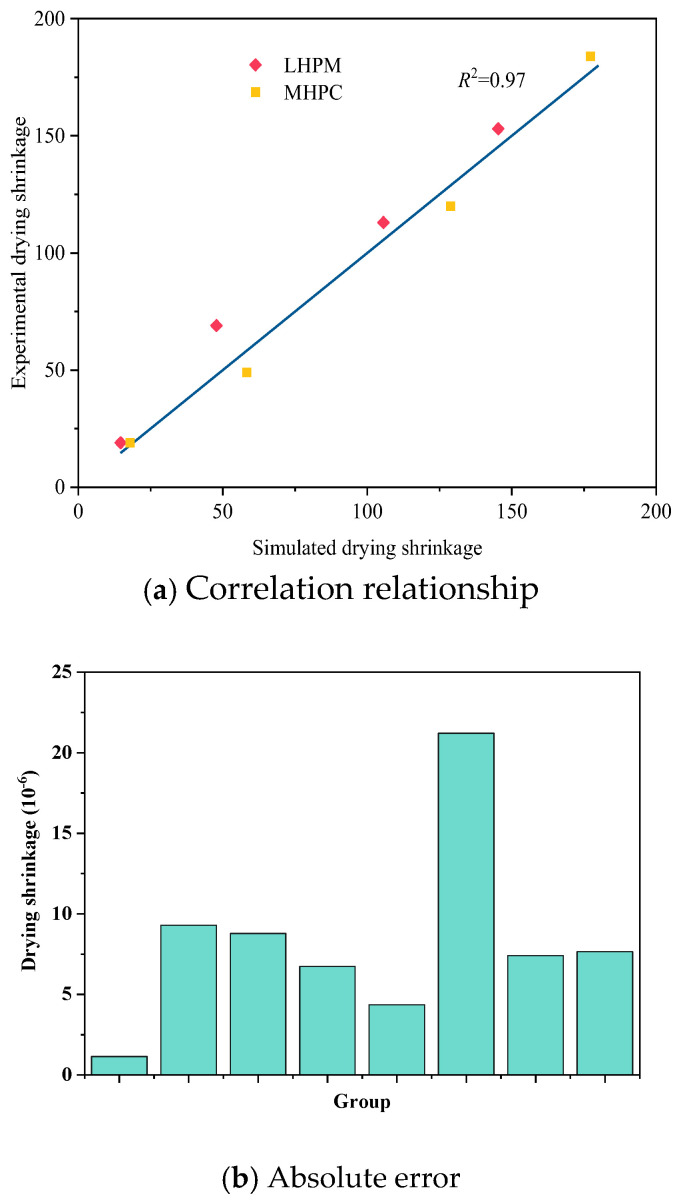
Correlation between results of experiment and simulation.

The concrete specimens are prepared with MHPC and LHPC and adopted at the engineering site. After hardening, the drying shrinkage of the concrete under the humidity environment *RH* = 0.5 was measured, as shown in Figure 8. It can be seen that the drying shrinkage of the concrete gradually increases with time at an early age, and the numerical results are consistent with the experimental results. For MHPC concrete, at 3 days, the drying shrinkage of MHPC is 19 × 10^−6^, increasing to 184 × 10^−6^ after 28 days. The numerical simulation results show that the drying shrinkage of MHPC is also 17.9 × 10^−6^ at 3 days and increases to 177.3 × 10^−6^ after 28 days, and the error is 3.66% compared with the experimental result. For LHPC concrete, the drying shrinkage in the experiment after 28 days is 153 × 10^−6^, and the drying shrinkage in numerical simulation after 28 days is 145.4 × 10^−6^, with an error of 4.99% compared with the experimental result. The results of two different kinds of concrete indicate that the proposed highly detailed simulation method can be employed to predict the early drying shrinkage of the concrete.

### 5.3. Influence of the Ambient Humidity on the Concrete Drying Shrinkage

**Figure 9:**
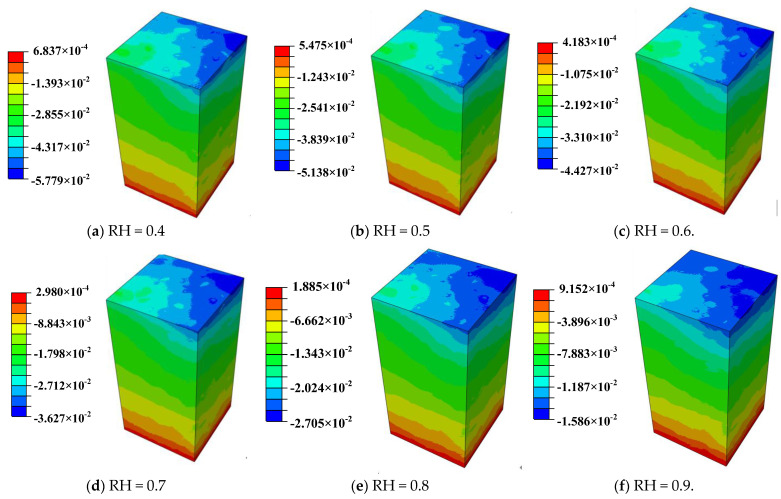
Concrete early drying shrinkage deformation under different ambient humidity.

MHPC concrete with an aggregate volume ratio of 0.52 is employed to analyze the early drying shrinkage deformation characteristics of concrete and the ambient humidity of concrete varying between 0.4 and 0.9. The changing trend of concrete drying shrinkage characteristics as a function of age was calculated and examined in the previous part. The early drying shrinkage deformation of concrete under different ambient humidity levels is shown in Figure 9. Due to water loss in three directions, the concrete corner experiences the most significant shrinkage deformation, while the middle part of the top surface experiences a gradual reduction in shrinkage deformation owing to water loss in one direction. For the massive concrete structures, the shrinkage deformation at the middle is a more representative parameter.

**Figure 10:**
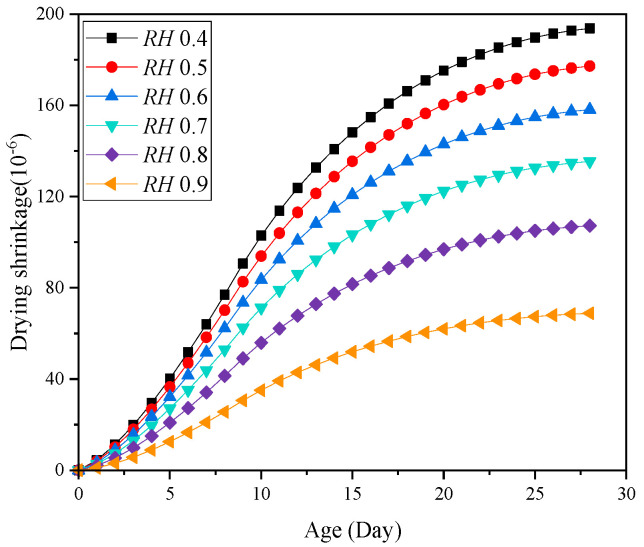
Concrete early drying shrinkage under different ambient humidity.

**Figure 11:**
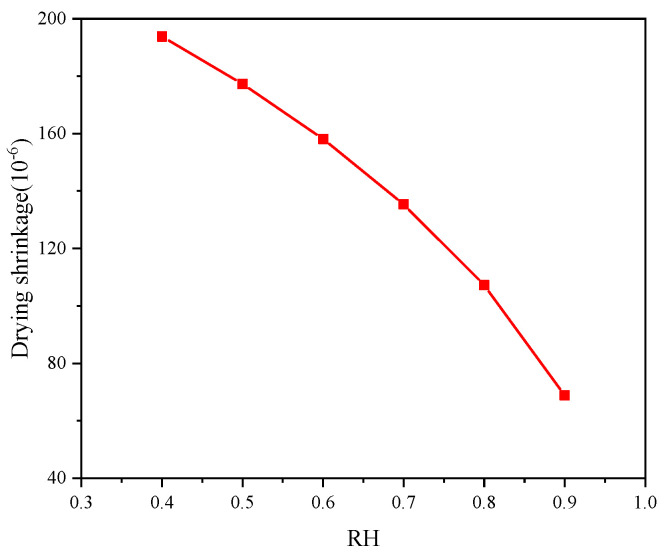
Drying shrinkage after 28 days with different ambient humidity.

**Figure 12:**
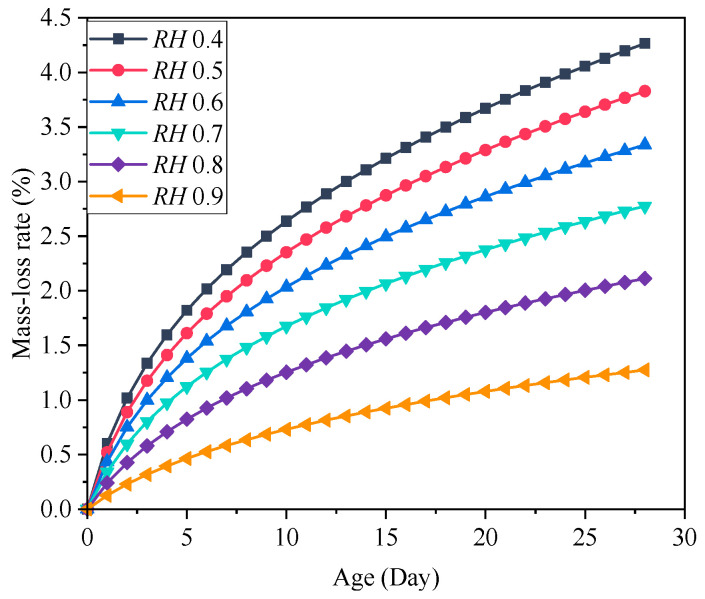
Concrete mass-loss rate under different ambient humidity.

**Figure 13:**
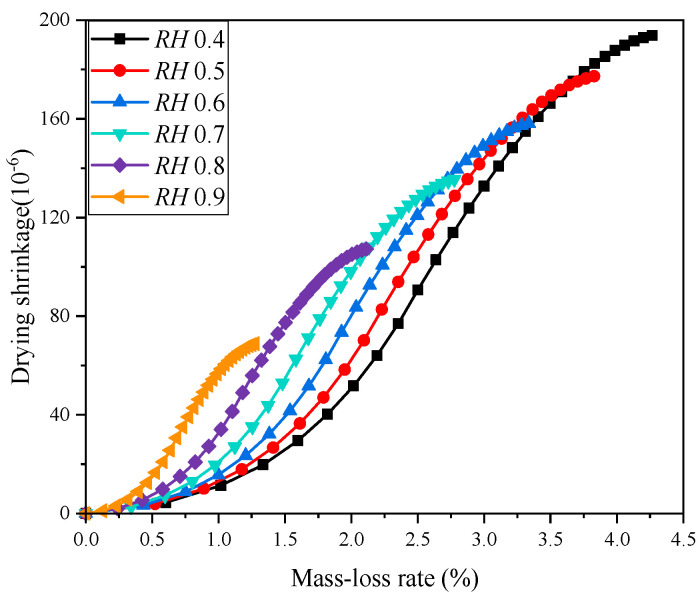
Relationship between drying shrinkage and mass-loss rate of concrete.

The concrete early drying shrinkage under different ambient humidity is shown in Figure 10. It shows that the concrete drying shrinkage increases gradually with the curing age, and the concrete early drying shrinkage decreases as the ambient humidity increases. The drying shrinkage after 28 days is shown in Figure 11. It can be found that the increase in ambient humidity will significantly reduce the early concrete drying shrinkage, which is consistent with the results acquired [22]. The concrete drying shrinkage decreases by 64.504% when the ambient humidity increases from 0.4 to 0.9. Figure 12 displays the concrete mass-loss rate under different ambient humidity. For concrete of the same age, the mass-loss rate decreases as ambient humidity increases [66]. At an ambient humidity of 0.4, the 28-day-old concrete experiences a mass-loss rate of 4.265%. While at an ambient humidity of 0.9, the same-age concrete experiences a mass-loss rate of 1.274%, which is 70.129% lower than that when the ambient humidity is 0.4. The relationship between concrete drying shrinkage and mass-loss rate under different ambient humidity is shown in Figure 13. It shows that the early concrete shrinkage is S-shaped, which can be divided into three stages. In the first stage, the concrete drying shrinkage increases slowly with the mass-loss rate, which is mainly due to insufficient hydration in the early stage. In the second stage, the concrete drying shrinkage increases linearly with the mass-loss rate. In the third stage, the concrete drying shrinkage is steady, which is consistent with the experimental results of Samouh et al. [16,17].

### 5.4. Influence of the Cement Type on the Concrete Drying Shrinkage

**Figure 14:**
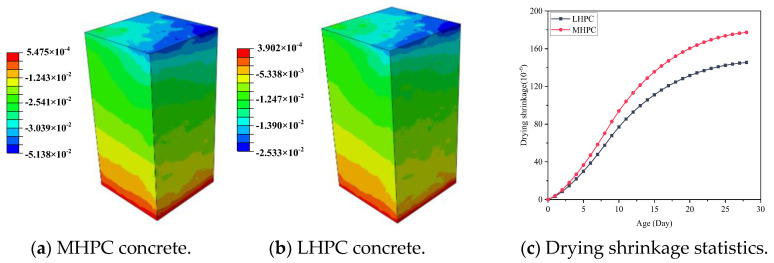
Early drying shrinkage with different types of cement.

**Figure 15:**
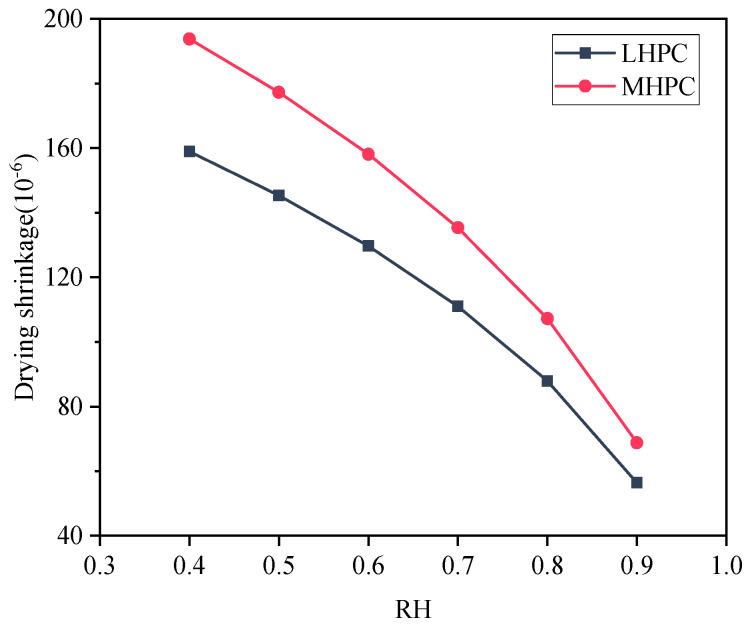
Drying shrinkage of 28-day-old concrete with different types of cement.

**Figure 16:**
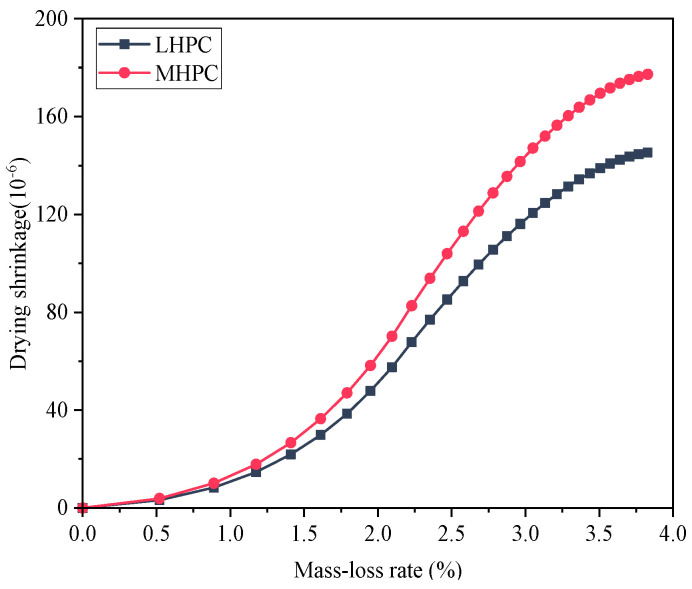
Relationship between the early drying shrinkage and mass-loss rate with different cements.

MHPC or LHPC is commonly utilized in hydraulic structures to efficiently decrease the rate of early hydration heat release of cement [46]. To compare early drying shrinkage between MHPC and LHPC concrete, the study focuses on concrete with an aggregate volume ratio of 0.52 [64,67]. Figure 14 presents the early drying shrinkage of MHPC and LHPC concrete. It can be found that MHPC concrete exhibits a higher early drying shrinkage compared to LHPC concrete, with the latter showing a drying shrinkage that is approximately 18.0% lower than the former after 28 days. The drying shrinkage of 28-day-old concrete with different types of cement is demonstrated in Figure 15. It can be seen that MHPC concrete experiences greater shrinkage than LHPC concrete after 28 days under the same ambient humidity. Additionally, Figure 16 shows the relationship between the early drying shrinkage and mass-loss rate of concrete based on MHPC and LHPC. The result indicates that the trend of early drying shrinkage of both the MHPC and LHPC exhibits three stages. The early drying shrinkage of MHPC concrete is greater than that of LHPC concrete at the same mass-loss rate during the drying process. Additionally, the LHPC concrete exhibits a smaller slope during the second stage and a lower peak value during the third stage compared to the MHPC concrete.

## 6. Case Study

**Figure 17:**
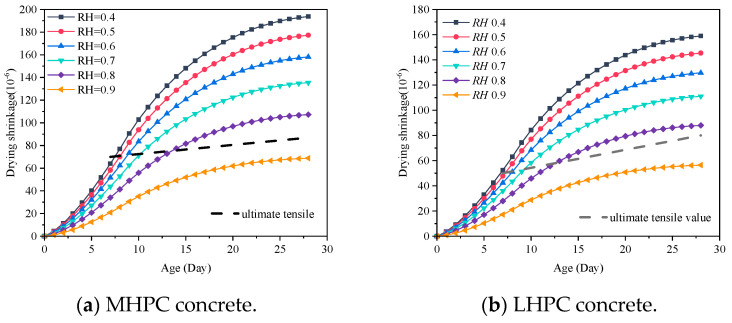
Ultimate tensile value and concrete early drying shrinkage.

The entrance to the gallery of the RCC dam in the northwest alpine region of China remains open after construction, resulting in the formation of a high-speed wind field in the gallery, leading to a significant decrease in ambient humidity. Inadequate curing conditions result in numerous vertical surface cracks on the side wall of the gallery, significantly affecting the safe operation of the RCC dam. The cracking time caused by different ambient humidity in the curing process is evaluated based on the in-situ ultimate tensile values of the concrete in this section, as shown in Figure 17. The result can provide guidance for similar massive concrete structures. The ultimate tensile value of the concrete is given in Table 4, and the volume ratio of aggregate is set equal to 0.52.

**Figure 18:**
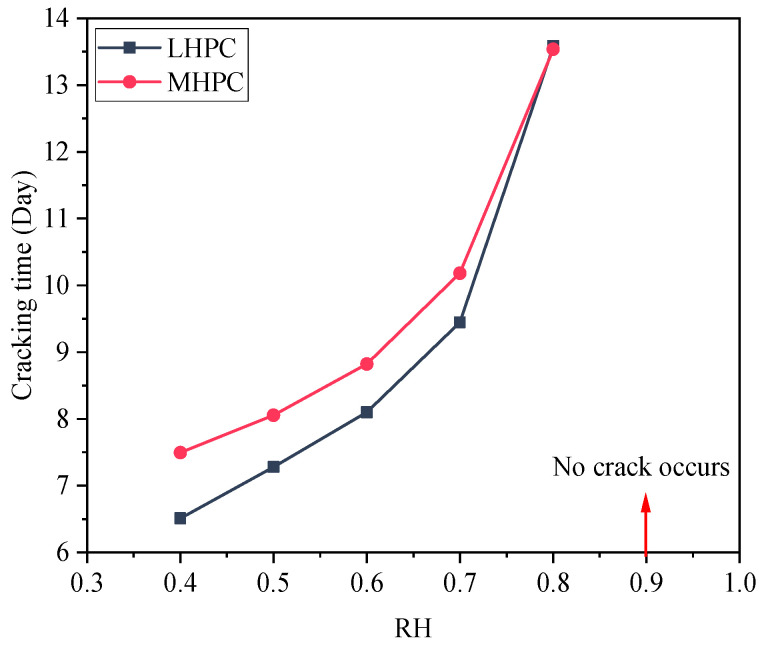
Cracking time of concrete under different ambient humidity.

According to the ultimate tensile value of concrete, the cracking time of concrete under different ambient humidity is calculated, as shown in Figure 18. The cracking time of MHPC concrete is later than that of LHPC concrete when the ambient humidity drops below 0.8. At an ambient humidity of 0.9, the MHPC and LHPC concrete exhibit no drying shrinkage crack at the early stage. The slope of cracking time for LHPC concrete surpasses that of MHPC concrete, indicating that LHPC concrete is more sensitive to ambient humidity. Additionally, the ambient humidity at which LHPC concrete does not crack is lower than that of MHPC concrete.

Based on the calculations above, it can be found that the optimal approach to minimize drying shrinkage cracks in massive concrete structures is to increase the ambient humidity during the curing process, e.g., by utilizing running water curing or surface moisture preservation measures to keep the ambient humidity over 0.9. The results can provide suitable strategies for the early maintenance of massive concrete structures and assist in the construction of crack-free dams in China.

## 7. Conclusions

This paper proposed an improved mesoscale model for the simulation of the early drying shrinkage of concrete. Based on this method, the influence of the ambient humidity, cement type, and aggregate volume ratio on the early drying shrinkage performance of concrete is studied. The results can be employed to examine the cracking time of the gallery in the RCC dam under different ambient humidity, thereby providing strategies for early curing measures of massive concrete structures and contributing to the construction of crack-free dams in China. The following results are obtained:

(1) The time-varying models of mortar shrinkage coefficient for the MHPC and LHPC concrete are established, and the relations for mortar shrinkage coefficient are calibrated according to the experiments on the mortar drying shrinkage. It is concluded that the drying shrinkage coefficient of the LHPC mortar is 0.82 times that of the MHPC mortar.
(2) The concrete humidity gradually decreased from the outside surface to the inside, and the surface humidity dropped faster than the internal. The humidity influence range of the concrete specimen is within 15 mm of the surface depth in the early drying shrinkage process.
(3) The early drying shrinkage of the concrete gradually decreases with the increase of ambient humidity or aggregate volume ratio, and the drying shrinkage of the LHPC concrete was apparently lower than that of the MHPC concrete. Therefore, the early drying shrinkage can be controlled by increasing ambient humidity via the LHPC or raising the aggregate volume ratio.
(4) Based on the calculation of the cracking time of the RCC dam gallery in the alpine region, it is found that the LHPC concrete is more sensitive to ambient humidity. When the ambient humidity is 0.9, the MHPC and LHPC concrete exhibit no drying shrinkage crack in the RCC dam gallery.

This paper presents a refined simulation method to investigate the early-age drying shrinkage behavior of concrete under varying environmental humidity, cement types, and aggregate volume ratios, providing valuable insights for the construction of RCC dams. In future work, we will aim to transcend the limitations from materials to structures, coupling the hydration heat of concrete, complex environmental factors, and structural loading conditions to provide broader applicability and guidance for the cracking control of various engineering practices.

## Figures and Tables

**Table 1:** Main chemical compositions of cement (%).

Cement Type	CaO	SiO_2_	Al_2_O_3_	Fe_2_O_3_	MgO	SO_3_	R_2_O *	Loss on Ignition
MHPC	60.3	21.7	4.3	4.8	4.9	1.9	0.4	0.9
LHPC	58.7	22.8	4.3	4.3	4.2	3.0	0.3	1.6

* Note: R_2_O is equivalent alkali content, R_2_O = Na_2_O + 0.658 K_2_O.

**Table 2:** Drying shrinkage experiment results of mortars (%).

Cement Type	0 Day	3 Days	7 Days	9 Days	14 Days	28 Days
MHPC	0	0.0132	0.0338	0.0448	0.0594	0.0744
LHPC	0	0.0143	0.0256	0.0345	0.0472	0.0615

**Table 3:** Properties in the mesoscale simulation model.

Parameters		Elastic Modulus (GPa)		Poisson’s Ratio	*D*	$\alpha_{sh}$
		**E7d**	**E28d**			
MHPC	Motar	18.90	31.60	0.22	Equation (5)	Equation (8)
	ITZ	15.12	25.28	0.20	10 Equation (5)	2.28 Equation (8)
	Aggregate	50		0.20	$\frac{\mathrm{Equation}\;\left(5\right)}{50}$	0
LHPC	Motar	20.30	27.80	0.22	Equation (5)	Equation (9)
	ITZ	16.24	22.24	0.20	10 Equation (5)	2.28 Equation (9)
	Aggregate	50		0.20	$\frac{\mathrm{Equation}\;\left(5\right)}{50}$	0

**Table 4:** Ultimate tensile values of the concrete.

Cement Type	Ultimate Tensile Value (10^−6^)	
	7 Days	28 Days
MHPC	70	87
LHPC	50	80

## Data Availability

The original contributions presented in this study are included in the article. Further inquiries can be directed to the corresponding author.

## References

[1] Nguyen Q., Afroz S., Zhang Y., Kim T., Li W., Castel A. (2022). Autogenous and total shrinkage of limestone calcined clay cement (LC3) concretes. Constr. Build. Mater..

[2] Shahmirzadi M., Gholampour A., Kashani A., Ngo T.D. (2021). Shrinkage behavior of cementitious 3D printing materials: Effect of temperature and relative humidity. Cem. Concr. Compos..

[3] Azarhomayun F., Haji M., Kioumarsi M., Shekarchia M. (2022). Effect of calcium stearate and aluminum powder on free and restrained drying shrinkage, crack characteristic and mechanical properties of concrete. Cem. Concr. Compos..

[4] Qin R., Hao H., Rousakis T., Lau D. (2019). Effect of shrinkage reducing admixture on new-to-old concrete interface. Compos. Part B Eng..

[5] Wu L., Farzadnia N., Shi C., Zhang Z., Wang H. (2017). Autogenous shrinkage of high performance concrete: A review. Constr. Build. Mater..

[6] Silva R., De Brito J., Dhir R. (2015). Prediction of the shrinkage behavior of recycled aggregate concrete: A review. Constr. Build. Mater..

[7] Zhang H., Wang Y., Lehman D., Geng Y., Kuder K. (2020). Time-dependent drying shrinkage model for concrete with coarse and fine recycled aggregate. Cem. Concr. Compos..

[8] Wang Q., Geng Y., Wang Y., Zhang H. (2020). Drying shrinkage model for recycled aggregate concrete accounting for the influence of parent concrete. Eng. Struct..

[9] Chen Y., Wei J., Huang H., Jin W., Yu Q. (2018). Application of 3D-DIC to characterize the effect of aggregate size and volume on non-uniform shrinkage strain distribution in concrete. Cem. Concr. Compos..

[10] Zhan P., He Z. (2019). Application of shrinkage reducing admixture in concrete: A review. Constr. Build. Mater..

[11] Liu L., Fang Z., Huang Z., Wu Y. (2022). Solving shrinkage problem of ultra-high-performance concrete by a combined use of expansive agent, super absorbent polymer, and shrinkage-reducing agent. Compos. Part B Eng..

[12] Xie T., Fang C., Ali M., Visintin P. (2018). Characterizations of autogenous and drying shrinkage of ultra-high performance concrete (UHPC): An experimental study. Cem. Concr. Compos..

[13] Wu Z., Shi C., Khayat K. (2019). Investigation of mechanical properties and shrinkage of ultra-high performance concrete: Influence of steel fiber content and shape. Compos. Part B Eng..

[14] Xia D., Song N., Li B., Zheng Y., Guo W., Wu J., Wang S. (2024). Understanding the synergetic effect of SAP and nano-silica on the mechanical properties, drying shrinkage and microstructures of alkali-activated slag/fly ash-based concrete. Constr. Build. Mater..

[15] Yang Y., Yue X., Chen B., Yang W., Guo W., Wang H. (2024). Autogenous shrinkage and cracking of ultra-high-performance concrete with soda residue as an internal curing agent. Mater. Struct..

[16] Samouh H., Rozière E., Loukili A. (2017). The differential drying shrinkage effect on the concrete surface damage: Experimental and numerical study. Cem. Concr. Res..

[17] Samouh H., Roziere E., Loukili A. (2019). Experimental and numerical study of the relative humidity effect on drying shrinkage and cracking of self-consolidating concrete. Cem. Concr. Res..

[18] Su H., Hu J., Li H. (2018). Multi-scale performance simulation and effect analysis for hydraulic concrete submitted to leaching and frost. Eng. Comput..

[19] Nariman N.A., Husek M., Ramadan A.M. (2022). Surrogate models for the damage responses of a reinforced concrete beam under explosive charges utilizing coupled finite element–stochastic methods. Eng. Comput..

[20] Deng Y., Shi X., Zhang Y., Chen J. (2021). Numerical modelling of rutting performance of asphalt concrete pavement containing phase change material. Eng. Comput..

[21] Idiart A., Bisschop J., Caballero A., Lura P. (2012). A numerical and experimental study of aggregate-induced shrinkage cracking in cementitious composites. Cem. Concr. Res..

[22] Havlásek P., Jirásek M. (2016). Multiscale modeling of drying shrinkage and creep of concrete. Cem. Concr. Res..

[23] Tang S., Wang S., Ma T., Tang C., Bao C., Huang X., Zhang H. (2016). Numerical study of shrinkage cracking in concrete and concrete repair systems. Int. J. Fract..

[24] Bolander J., Berton S. (2004). Simulation of shrinkage induced cracking in cement composite overlays. Cem. Concr. Compos..

[25] Ožbolt J., Balabanić G., Periškić G., Kušter M. (2010). Modelling the effect of damage on transport processes in concrete. Constr. Build. Mater..

[26] Ožbolt J., Balabanić G., Kušter M. (2011). 3D Numerical modelling of steel corrosion in concrete structures. Corros. Sci..

[27] Ožbolt J., Oršanić F., Balabanić G., Kušter M. (2012). Modeling damage in concrete caused by corrosion of reinforcement: Coupled 3D FE model. Int. J. Fract..

[28] Ožbolt J., Oršanić F., Balabanić G. (2016). Modeling influence of hysteretic moisture behavior on distribution of chlorides in concrete. Cem. Concr. Compos..

[29] Abdelatif A.O., Ožbolt J., Gambarelli S. (2018). 3D finite element modelling of corrosion of lap splice joints in concrete. Constr. Build. Mater..

[30] Ožbolt J., Gambarelli S., Zadran S. (2022). Coupled hygro-mechanical meso-scale analysis of long-term creep and shrinkage of concrete cylinder. Eng. Struct..

[31] Li W., Zhao Y., Zhang Y., Xie Z., Zhang J., Huang F., Meng L., He Z., Xia J., Zhang Y. (2024). Optimized mix design method of ultrahigh performance concrete (UHPC) and effect of high steel fiber content: Mechanical performance and shrinkage properties. J. Build. Eng..

[32] Jin L., Liu M., Zhang R., Du X. (2020). 3D meso-scale modelling of the interface behavior between ribbed steel bar and concrete. Eng. Fract. Mech..

[33] Zhang J., Zhang M., Dong B., Ma H. (2022). Quantitative evaluation of steel corrosion induced deterioration in rubber concrete by integrating ultrasonic testing, machine learning and mesoscale simulation. Cem. Concr. Compos..

[34] Stamati O., Roubin E., Andò E., Malecot Y. (2018). Phase segmentation of concrete X-ray tomographic images at meso-scale: Validation with neutron tomography. Cem. Concr. Compos..

[35] Huang Y., Yan D., Yang Z., Liu G. (2016). 2D and 3D homogenization and fracture analysis of concrete based on in-situ X-ray Computed Tomography images and Monte Carlo simulations. Eng. Fract. Mech..

[36] Huang Y., Yang Z., Ren W., Liu G., Zhang C. (2015). 3D meso-scale fracture modelling and validation of concrete based on in-situ X-ray Computed Tomography images using damage plasticity model. Int. J. Solids Struct..

[37] Li Y., Ruan X., Akiyama M., Zhang M., Xin J., Lim S. (2021). Modelling method of fibre distribution in steel fibre reinforced concrete based on X-ray image recognition. Compos. Part B Eng..

[38] Thilakarathna P., Baduge K., Melndis P., Vimonsatit V., Lee H. (2020). Mesoscale modelling of concrete—A review of geometry generation, placing algorithms, constitutive relations and applications. Eng. Fract. Mech..

[39] Peng R., Qiu W., Teng F. (2020). Three-dimensional meso-numerical simulation of heterogeneous concrete under freeze-thaw. Constr. Build. Mater..

[40] Zhang H., Sheng P., Zhang J., Ji Z. (2019). Realistic 3D modeling of concrete composites with randomly distributed aggregates by using aggregate expansion method. Constr. Build. Mater..

[41] Guo J., Zhang J., Yu H., Ma H., Wu Z. (2022). Experimental and 3D mesoscopic investigation of uniaxial compression performance on basic magnesium sulfate cement-coral aggregate concrete (BMSC-CAC). Compos. Part B Eng..

[42] Xiong X., Xiao Q. (2021). Meso-scale simulation of bond behaviour between retarded-bonded tendons and concrete. Eng. Struct..

[43] Yu Y., Zheng Y., Zhao X. (2021). Mesoscale modeling of recycled aggregate concrete under uniaxial compression and tension using discrete element method. Constr. Build. Mater..

[44] Wang P., Gao N., Ji K., Stewart L., Arson C. (2020). DEM analysis on the role of aggregates on concrete strength. Comput. Geotech..

[45] Fu Q., Bu M., Zhang Z., Xu W., Yuan Q., Niu D. (2023). Hydration characteristics and microstructure of alkali-activated slag concrete: A review. Engineering.

[46] Wang L., Yang H., Zhou S., Chen E., Tang S. (2018). Mechanical properties, long-term hydration heat, shinkage behavior and crack resistance of dam concrete designed with low heat Portland (LHP) cement and fly ash. Constr. Build. Mater..

[47] Kim J.K., Lee C.S. (1999). Moisture diffusion of concrete considering self-desiccation at early ages. Cem. Concr. Res..

[48] Huang H., Garcia R., Huang S.S., Guadagnini M., Pilakoutas K. (2019). A practical creep model for concrete elements under eccentric compression. Mater. Struct..

[49] Jiang Z., Sun Z., Wang P. (2006). Internal relative humidity distribution in high-performance cement paste due to moisture diffusion and self-desiccation. Cem. Concr. Res..

[50] Wang Y. (2017). Study on long-term behavior of long span concrete arch bridge with stiffed concrete filled steel tube in natural environment. Southwest Jiaotong Univ..

[51] Zhao L., Wang P., Wang L., Zhao T., Guang W. (2021). Analysis of parameters for temperature and humidity response in concrete: Moisture diffusion coefficient and thermal conductivity. Mater. Rev..

[52] Li S., Li Q. (2011). Two dimensional analysis of drying shrinkage micro-cracking in concrete with modified smeared cracking model. Eng. Mech..

[53] Jin H., Zhou Y. (2022). Study of three-dimensional concrete shrinkage crack behavior using a mesoscale concrete model with actual geometry and random aggregate. Sci. Sin. (Technol.).

[54] (2004). Standard Test Method for Drying Shrinkage of Motar.

[55] (2019). Standard Test Method for Autogenous Strain of Cement Paste and Mortar.

[56] Yang Z., Ren W., Sharma R., McDonald S., Mostafavi M., Vertyagina Y., Marrow T.J. (2017). In-situ X-ray computed tomography characterisation of 3D fracture evolution and image-based numerical homogenisation of concrete. Cem. Concr. Compos..

[57] Jin L., Yu W., Du X., Yang W. (2019). Mesoscopic numerical simulation of dynamic size effect on the splitting-tensile strength of concrete. Eng. Fract. Mech..

[58] Naderi S., Zhang M. (2021). Meso-scale modelling of static and dynamic tensile fracture of concrete accounting for real-shape aggregates. Cem. Concr. Compos..

[59] Li M., Zhang M. (2018). Primary investigation of isogeometric analysis for hydraulic structures numerical simulation. J. Hydraul. Eng..

[60] Zhang M., Li M., Shen Y., Zhang J. (2019). Isogeometric shape optimization of high RCC gravity dams with functionally graded partition structure considering hydraulic fracturing. Eng. Struct..

[61] Maleki M., Rasoolan I., Khajehdezfuly A., Jivkov A.P. (2020). On the effect of ITZ thickness in meso-scale models of concrete. Constr. Build. Mater..

[62] Chen H., Xu B., Wang J., Zhou T., Nie X., Mo Y. (2020). Parametric analysis on compressive strain rate effect of concrete using mesoscale modeling approach. Constr. Build. Mater..

[63] Jin L., Jiang X., Xia H., Chen F., Du X. (2020). Size effect in shear failure of lightweight concrete beams wrapped with CFRP without stirrups: Influence of fiber ratio. Compos. Part B Eng..

[64] Li M., Min Q., Zhang M., Liu D., Shen Y., Feng D. (2022). Mechanical experiments and precise simulation of a novel functional material: Electrically conductive roller-compacted concrete. Sci. Sin. (Technol.).

[65] Wang J., Li H., Ma C., Cai C., Wang J. (2024). Effect of surface curing condition on the humidity field and moisture transfer in concrete. Constr. Build. Mater..

[66] Zhang J., Wang J.H., Han Y.D. (2015). Simulation of moisture field of concrete with pre-soaked lightweight aggregate addition. Constr. Build. Mater..

[67] Wang L., Yang H.Q., Dong Y., Chen E., Tang S.W. (2018). Environmental evaluation, hydration, pore structure, volume deformation and abrasion resistance of low heat Portland (LHP) cement-based materials. J. Clean. Prod..

